# Endoscopic submucosal dissection versus transanal local excision for rectal carcinoid: a comparative study

**DOI:** 10.1186/s12957-016-0923-4

**Published:** 2016-06-21

**Authors:** Fei-hu Yan, Zheng Lou, Shi-jie Hu, Xiao-dong Xu, Hao Wang, Han-tao Wang, Rong-gui Meng, Chuan-gang Fu, Wei Zhang, Jian He, En-da Yu

**Affiliations:** Department of Colorectal Surgery, Changhai Hospital, Second Military Medical University, Changhai Road 168#, Shanghai, China; Department of General Surgery, 413 Hospital, Zhoushan, China; Department of Colorectal Surgery, Jianmin Colorectal Disease Hospital, Yong gang South Road 225#, Ningbo, China

**Keywords:** Rectal carcinoid, Endoscopic submucosal dissection, Transanal local excision

## Abstract

**Aim:**

The aim of this study is to compare the short-term clinical outcomes between endoscopic submucosal dissection and transanal local excision for rectal carcinoid tumors.

**Methods:**

Between 2007 and 2012, 31 patients with rectal carcinoid underwent endoscopic submucosal dissection at our hospital. They were compared with a matched cohort of 23 patients who underwent transanal local excision for rectal carcinoid between 2007 and 2012. Short-term clinical outcomes including surgical parameters, postoperative recovery, and oncologic outcomes were compared between the two groups.

**Results:**

The mean size of tumors was significantly bigger in the transanal local excision group (0.8 ± 0.2 versus 1.1 ± 0.5 cm; *P* = 0.018). En bloc resection was achieved for 30 patients (97 %) in the endoscopic submucosal dissection group and all the patients in the transanal local excision group. The operation time was longer in the transanal local excision than that in the endoscopic submucosal dissection group (40.0 ± 22.7 min versus 12.2 ± 5.3 min; *P* < 0.001). Complications in the transanal local excision group were five cases of acute retention of urine. There was no local recurrence or distant metastasis in either group during the follow-up period.

**Conclusion:**

For the treatment of rectal carcinoid tumors with diameter <1 cm, endoscopic submucosal dissection has better short-term clinical outcomes than transanal local excision in terms of faster recovery and possibly a lower morbidity rate. Transanal local excision may be the first therapeutic choice of scar-embedded rectal carcinoid tumors.

## What does this paper add to the literature statement?

In this work, we compare the short-term clinical outcomes between endoscopic submucosal dissection (ESD) and transanal local excision (TALE) for rectal carcinoid tumors, so as to help surgeons decide which method should be chosen for these patients.

## Background

Rectal carcinoid tumors account for 18.5 % of all carcinoid tumors. Although these tumors represent only 1.3 % of all rectal neoplasms, their incidence is drastically increasing [1, 2]. These tumors can show a broad range of clinical behavior, from benign and asymptomatic to disseminated and metastatic [3]. Because rectal carcinoid tumors often present as small and localized tumors that rarely recur after resection, rectal carcinoid tumors have the best prognosis among all carcinoid tumors with a 5-year survival rate at 88.3 %, and thus have usually been managed with local excision [4–6].

For rectal carcinoid tumors not amenable to conventional colonoscopic removal, the alternative treatment option is TALE procedure. This local procedure has proved to be safe and effective in the treatment of low rectal lesions. However, TALE approach must be performed with the patient under anesthesia, such as spinal anesthesia, general endotracheal anesthesia (GETA), and IV sedation + local depending on tumor location and size, especially the characteristics and comorbidities of patients.

ESD is a novel colonoscopic method that enables en bloc resection for colorectal lesions. It can be performed with patients without anesthesia. The ESD approach has a reported perforation rate of 2 to 10 %, an en bloc resection rate of 80 to 90 %, and a short-term recurrence rate for colorectal ESD of 0 to 2.1 % [7].

In the traditional sense, the indications of TALE for rectal carcinoid tumors are as follows: (1) tumor located less than 7 cm to anal edge, (2) tumor size accounted less than 1/3 lumen diameter, (3) tumor node metastasis (TNM) staged earlier than T1. Except the above situation, choose surgical oncologic resection. Despite a growing understanding of the clinical effectiveness of local excision for small rectal carcinoid tumors, there is still controversy concerning the best surgical choice for rectal carcinoid tumors with different diameters [8]. The aim of this study is to report our experiences of adopting two different local excision procedures according to tumor diameter. To our knowledge, there is no report about the surgical strategies and results of ESD and TALE for rectal carcinoid tumors.

## Methods

### Patients and study design

Between October 2007 and December 2012, 78 patients with rectal carcinoid were admitted to Changhai Hospital. We included 54 patients in our study (31 patients underwent ESD, 23 patients underwent TALE). The inclusion criteria for TALE and ESD based upon tumor size, location, and TNM stage: (1) tumor located less than 7 cm to anal edge, (2) tumor size accounted less than 1/3 lumen diameter, (3) TNM staged earlier than T1. Then, we excluded 24 patients who underwent surgical oncologic resection. The clinical data of patients with rectal carcinoid who underwent either ESD or TALE were retrospectively reviewed. In this study, we enrolled patients with rectal carcinoid tumors that did not show regional lymph node enlargement on computed tomography (CT) scanning or endorectal ultrasonography (EUS) as previously reported [9]. Patients underwent local excision using ESD or TALE. All specimens were referred to pathologists and examined microscopically for tumor size, depth of invasion, and resection margin status. The patients’ characteristics, operative details, pathological results, and short-term outcomes were recorded. Six experienced, qualified surgeons and endoscopists (En-da Yu, 30 years experience; Zheng Lou, 12 years experience; Rong-gui Meng, 32 years experience; Wei Zhang, 25 years experience; Lian-jie Liu, 26 years experience; Li-qiang Hao, 19 years experience, et al.) performed the procedures (TALE and ESD).

Informed consent was obtained from all patients. The study protocol conformed to the ethical guidelines of the Helsinki Declaration. The ethical review has been approved by The Ethics Committee of Changhai Hospital.

### Data and materials

#### TALE

In our study, all TALE procedures were performed with the patient under spinal anesthesia, lithotomy position, or clasp knife position. Anal retractors were first introduced into the anal canal to maintain exposure. Normal saline was injected into the submucosal plane with an injector syringe to create a visible submucosal cushion for elevation of the lesion. The tumor then was excised with electrocautery or an ultrasonic knife (Johnson) under direct vision, performed in a submucosal resection, then the wound was closed with absorbable sutures (Johnson). But, one patient underwent additional surgery (TALE) after ESD because the ESD specimen showed a positive resection margin, in a full thickness fashion.

#### ESD

In our center, we use a two-person approach to colonoscopy. ESD was performed using a conventional endoscope (CF-H260, Olympus, Tokyo, Japan). A mixture of glycerin and fructose, normal saline, adrenaline, and methylene blue was injected into the submucosal plane with a submucosal injection needle (Alton) to create a visible submucosal cushion for elevation of the lesion. Mucosal incision and submucosal dissection were performed using the needle knife or insulated tip knife (Olympus Endoscopy Medical System, Tokyo, Japan) depending on the individual endoscopists’ preference. All procedures were performed without anesthesia. The patients undergoing ESD did not receive any kind of IV sedation.

### Outcome measurements

Short-term clinical outcomes included operative time, morbidity rate, time to ambulation, and hospital stay. Morbidity was defined as any complication that required reintervention or resulted in prolonged hospital stay. Bleeding was defined as any bleeding episode during or after the operation that required blood transfusion. Perforation was either identified during the operation or diagnosed when free intraperitoneal gas was found on abdominal imaging after the operation. Acute retention of urine was defined as failure to void within 24 h after the operation. Other operative details including the operative time and the en bloc resection rate also were compared between the two groups.

### Methods of postoperative surveillance

We mainly used medical records and nursing records to get initial postoperative information; we also conformed these information by telephone follow-up and got more follow-up data.

### Statistical analysis

Continuous variables were presented as mean (standard deviation), and dichotomous variables were presented as number and percentage values. Patients with different procedures were compared in regard to age, sex, and pathologic tumor characteristics with Fisher exact test, chi-square test, or independent *t* test, as appropriate. All analyses were performed with SPSS version 17 statistical software package (SPSS, Inc., Chicago, IL).

## Results

The baseline characteristics of the two groups were shown in Table 1. The two groups did not differ in terms of age, sex, or distance from anal verge. The mean size of tumors was significantly bigger in the TALE group (0.8 ± 0.2 versus 1.1 ± 0.5 cm; *P* = 0.018). En bloc resection was achieved for 30 patients (97 %) in the ESD group and all the patients in the TALE group.

**Table 1:** Demographic data and tumor characteristics for the TALE and ESD groups

	*TALE* (*n = 23*)	*ESD* (*n = 31*)	*p* value
Mean age (years)	47.9 ± 11.7	52.2 ± 10.2	0.159
Sex (male/female)	14/9	22/9	0.436
Tumor size (cm)	1.1 ± 0.5	0.8 ± 0.2	0.018
Distance from anal verge (cm)	5.4 ± 1.5	5.9 ± 2.3	0.435
En bloc resection: *n* (%)	23 (100 %)	30 (97 %)	1.0
Operation time	40.0 ± 22.7	12.2 ± 5.3	<0.001
Muscular invasion	0	0	/
Lymphovascular invasion	0	0	/

Operative time was longer in the TALE than in the ESD group (40.0 ± 22.7 min versus 12.2 ± 5.3 min; *P* < 0.001). Complications in the TALE group were five cases of AROU. There were no bleeding or perforation cases in either group. Table 2 summarizes procedure-related parameters.

**Table 2:** Short-term postoperative complications

Procedure	*AROU*	*Anus ache*	*Bleeding*	*Perforation*
TALE	5	16	0	0
ESD	0	3	0	0
*p* value	0.024	<0.001	/	/

Follow-up data was available in 24 patients in the ESD group because seven patients were not due for follow-up at the time of analysis. One patient underwent additional surgery (TALE) after ESD because the ESD specimen showed positive resection margin. Follow-up data was analyzed in 18 patients in the TALE group. The median follow-up period was 16.4 months (range 8–31 months) in the ESD group and 28.4 months (range 8–68 months) in the TALE group. The median number of follow-up colonoscopies was 1.9 times (range 1–4 times) in the ESD group and 2.5 times (range 1–5 times) in the TALE group. There was no local recurrence or distant metastasis in any patients in either group during the follow-up period.

## Discussion

Rectal carcinoid tumor is a rare condition accounting for only 1.3 % of the overall rectal tumor cases [10, 11]. Tumor size has been a known prognostic factor for rectal carcinoid tumors. ESD, TALE, and radical resection have been performed as treatments of rectal carcinoid tumor. However, it is currently difficult to decide between treatments such as local excision and radical surgery, especially for tumors that are >1 to ≤2 cm in diameter. It appears that small rectal carcinoids <1 cm in size can be safely managed by local excision. For tumors >1 to ≤2 cm, local excision is usually recommended, but radical surgery should be considered if there is evidence of lymph node metastasis [12]. The preoperative radiological evaluation of lymph node metastasis was critical. Pelvic imaging evaluation such as CT or endorectal ultrasound was made, and no enlarged lymph nodes were found in our patients before surgery.

ESD and TALE are performed for local excision of rectal carcinoids in daily clinical work. Which is the best method for the local resection of rectal carcinoids? Son et al. reported pathologically determined complete-resection (P-CR) rates for small rectal carcinoid tumors excised by using several methods. The P-CR rates were 30.9, 72.0, and 81.8 % for a conventional endoscopic polypectomy, ESD, and TALE [13]. TALE is a traditional procedure for treating lower rectal tumors and provides deeper vertical resection margin. Kim et al. [14] described the complete-resection rate for transanal surgery as over 97 %. Recently Son et al. reported that the pathologically complete-resection rates were 30.9, 72.0, and 81.8 % for conventional polypectomy, advanced endoscopic techniques, and surgical local excision, respectively. Pathologically complete-resection of small rectal carcinoid tumors was more likely to be achieved when using surgical local excision [14]. Ono et al. [15] described results obtained with 14 rectal carcinoid tumors treated by ESD between 1999 and 2002. All tumors were completely resected, and no recurrence was noted at a median follow-up of 10.5 months. In our study, the mean tumor size was 1.1 ± 0.5 cm and 0.8 ± 0.2 cm in TALE and ESD group, respectively. All lesions were confined to the submucosal layer. The pathologically complete-resection rates were 97 and 100 % for ESD and TALE, respectively. The results suggested that pathologically complete resection of rectal carcinoid tumors can be achieved when using proper procedure according to the tumor size.

However, TALE must be also considered to be more invasive because of the risk associated with the use of anesthesia [14]. Spinal anesthesia is a risk factor for acute retention of urine (AROU). In fact, anal dilation or retraction because of operation may cause anal pain and also may result in AROU. These factors explain why the TALE group had a significantly higher incidence of AROU. On the other hand, ESD is performed without anesthesia, and there is no need to dilate the anal sphincter. This may account for the lower morbidity rate and shorter operative time in the ESD group.

But for a local recurrence case, TALE may be more appropriate. ESD was performed after a mixture of glycerin and fructose, normal saline, adrenaline, and methylene blue was injected into the submucosal layer in order to achieve en bloc resection and avoid perforation. However, the lesion cannot be lifted after injection because of the existence of fibrosis in the submucosal layer in the case of local recurrence. The cut and the dissection steps that use cutting devices may be technically difficult. Fujishiro et al. reported that endoscopic en bloc resection was not achieved in some colorectal tumors because of fibrosis which prevented a sufficient submucosal fluid cushion in the submucosal layer [16]. In the TALE group, one patient had a reoperation because of positive resection margin with ESD. By TALE procedure, the tumor was removed with R0 resection. From our opinion, TALE may be a safe and effective method in the treatment of scar embedded lesion.

This study had some limitations. First, this was a retrospective study. We were unable to fully evaluate possible confounders, owing to the retrospective nature of this study. The second limitation of this study was that further follow-up studies are required to more accurately define the long-term outcomes of TALE and ESD. The third limitation of this study was the limited presented data and the number of patients enrolled. It is because the disease incidence is low that there is a difficulty of achieving an adequate number of cases for comparative analysis. Best results should be achieved with work with more patients and preferably a multicenter.

## Conclusion

In conclusion, for the treatment of rectal carcinoid tumors with diameter <1 cm, ESD has better short-term clinical outcomes than TALE in terms of faster recovery and possibly a lower morbidity rate. TALE may be the first therapeutic choice of scar embedded rectal carcinoid tumors. Prospective studies with larger samples are needed to validate the benefits of TALE.

## Abbreviations

AROU, acute retention of urine; P-CR, pathologically determined complete resection; CT, computed tomography; EUS, endorectal ultrasonography; ESD, endoscopic submucosal dissection; TALE, transanal local excision; GETA, general endotracheal anesthesia; TNM, tumor node metastasis.
